# A hypopigmented and enlarging granulomatous eruption

**DOI:** 10.1016/j.jdcr.2024.02.035

**Published:** 2024-03-25

**Authors:** Michelle Sikora, Kristen Lo Sicco, Shane Meehan, Frank Martiniuk, William Levis, Avrom S. Caplan

**Affiliations:** a New York Medical College, Valhalla, New York; b The Ronald O. Perelman Department of Dermatology, New York University Grossman School of Medicine, New York, New York; c JME Group Associates Inc., Roseland, New Jersey; d Department of Dermatology, Director of New York Hansen's Disease Program, Bellevue Hospital Center, New York, New York

**Keywords:** borderline tuberculoid leprosy, Hansen’s disease, leprosy, paucibacillary leprosy

A patient in his 40s was referred for a 2-year history of hypopigmented plaques. Prior biopsies demonstrated “granulomatous dermatitis.” Systemic evaluations revealed no evidence of sarcoidosis. Topical and intralesional corticosteroids, prednisone, and hydroxychloroquine were trialed without improvement. On referral, examination revealed an anesthetic hypopigmented plaque on the right upper extremity (Fig 1) and a pink plaque on the chest (Fig 2). A 4-mm punch biopsy of the chest revealed a perivascular infiltrate of lymphocytes with loosely formed aggregates of epithelioid histiocytes and a subepidermal grenz zone (Fig 3). Acid-fast bacilli staining was negative. He reports moving from Guyana years ago.

**Fig 1:**
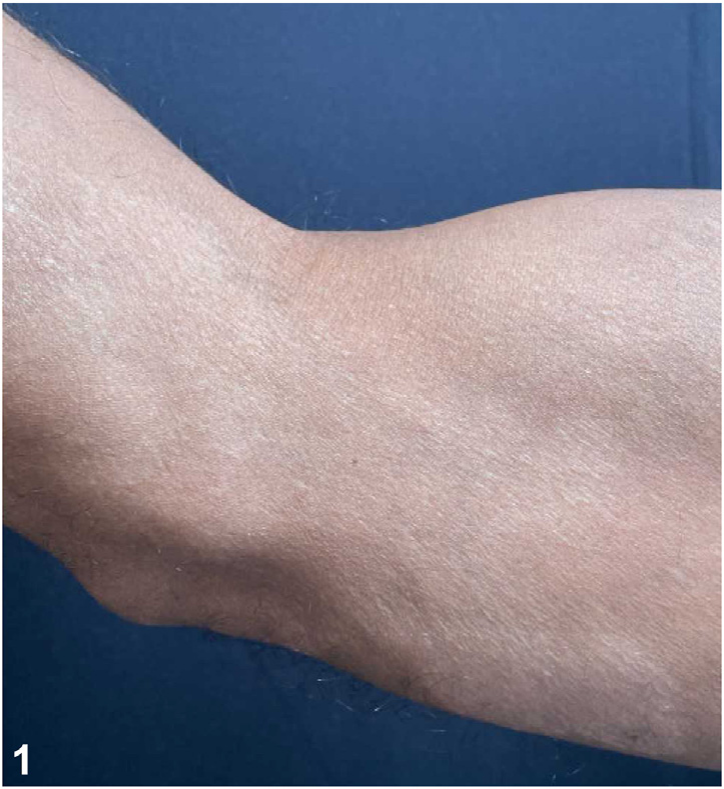

**Fig 2:**
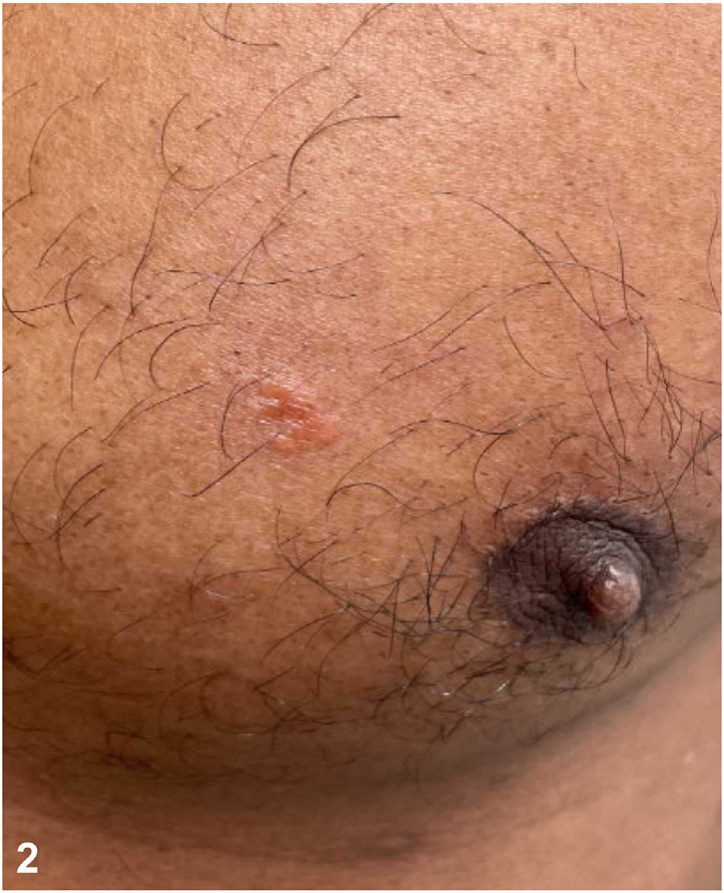

**Fig 3:**
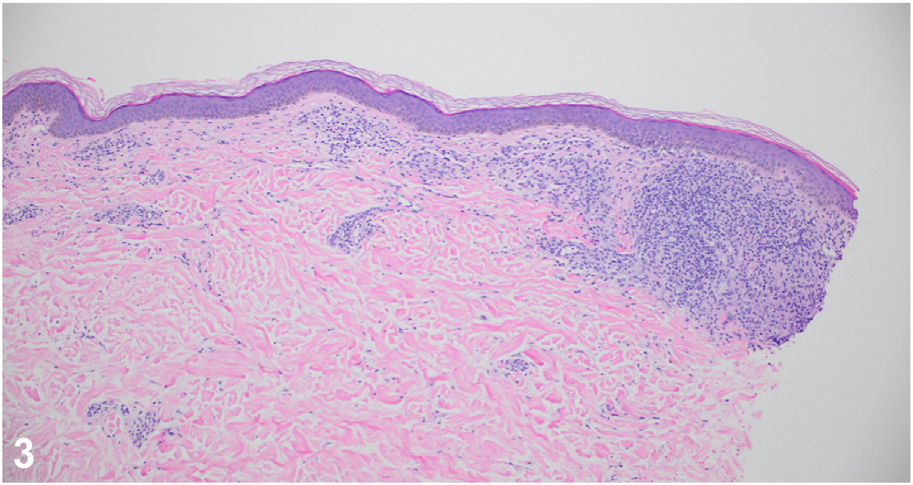

**Question 1: What is the most likely diagnosis?**

A. Cutaneous sarcoidosis
B. Mycosis fungoides
C. Borderline tuberculoid (BT) leprosy
D. Lepromatous leprosy (LL)
E. Granuloma annulare

**Answers:**

A. Cutaneous sarcoidosis – Incorrect. Although granulomas were revealed on biopsy, the diagnosis of “isolated cutaneous sarcoidosis” is controversial and alternative diagnoses should be considered. Given the anesthetic plaque, grenz zone, and immigration from a leprosy-endemic country, sarcoidosis is not the best answer.
B. Mycosis fungoides – Incorrect. Although mycosis fungoides can present with hypopigmented plaques, especially on the limbs or trunk in patients with skin of color,^1^ the histology is not consistent with mycosis fungoides. Furthermore, anesthetic plaques would be unexpected.
C. Borderline tuberculoid (BT) leprosy – Correct. This patient presented with scattered, well-defined, hypopigmented to pink, anesthetic skin patches, and moved to the United States from a leprosy-endemic country.^2^ The clinical presentation, history, and histopathology are consistent with BT leprosy by the Ridley-Jopling classification, which combines clinical manifestations, histology, and density of bacilli on smears (bacteriologic index) to categorize leprosy along a spectrum.^3^ BT generally corresponds to paucibacillary leprosy by World Health Organization (WHO) classifications, which is based on bacteriologic index where available or number of lesions.^3^
D. Lepromatous leprosy (LL) – Incorrect. The patient’s clinical presentation is consistent with BT leprosy. LL is characterized by an insufficient cell-mediated immune response leading to widespread lesions, high bacterial loads resulting in positive acid-fast bacilli staining, and stocking and glove distribution peripheral neuropathy.^2^ LL generally corresponds to multibacillary leprosy by WHO classifications.
E. Granuloma annulare – Incorrect. The histology and hypopigmented, anesthetic plaques are consistent with BT leprosy. Palisaded histiocytes and mucin deposition would characterize histopathology of granuloma annulare.

**Question 2: What is the most appropriate next step?**

A. Repeat biopsy and stains for acid-fast bacilli
B. Send serologic tests to confirm a diagnosis of leprosy
C. Initiate therapy with dapsone and rifampicin
D. Initiate therapy with dapsone
E. Initiate therapy with adalimumab

**Answers:**

A. Repeat biopsy and stains for acid-fast bacilli – Incorrect. A clinical diagnosis of BT leprosy was made given the anesthetic skin lesions in the setting of histopathology findings and the patient’s history. Furthermore, in BT leprosy, staining for acid-fast bacilli is often negative.
B. Send serologic tests to confirm a diagnosis of leprosy – Incorrect. No reliable blood tests are available for the diagnosis of leprosy.^3^^,^^4^ The serologic test Phenolic glycolipid is unavailable in the United States, and patients with BT leprosy are typically seronegative.^2^^,^^4^ Diagnostic approaches include skin biopsies, slit skin smears, and polymerase chain reaction.^2^^,^^3^ Slit skin smear provides a bacteriologic index to estimate acid-fast bacilli count, aiding in determining the diagnosis and classification of leprosy by WHO classifications.^3^
C. Initiate therapy with dapsone and rifampicin – Correct. The National Hansen’s Disease Program recommends a 12-month regimen of dapsone and rifampicin for immunocompetent adults with BT leprosy, whereas the WHO recommends a 6-month regimen involving dapsone, rifampicin, and clofazimine.^2^^,^^3^
D. Initiate therapy with dapsone – Incorrect. Multidrug therapy is a cornerstone of both the WHO and National Hansen’s Disease Program treatment guidelines for leprosy. While dapsone is a component of therapy, single drug regimens are inappropriate in treating leprosy.^4^
E. Initiate therapy with adalimumab – Incorrect. Adalimumab, a tumor necrosis factor-alpha inhibitor, is not a treatment for BT leprosy. tumor necrosis factor-alpha inhibitor use may be a risk factor for developing leprosy or reactivating subclinical infection.^5^

**Question 3: This patient is at risk for?**

A. A type 1 reversal reaction
B. A type 2 reversal reaction
C. Lucio Phenomenon
D. Drug resistance
E. None of the above

**Answers:**

A. A type 1 reversal reaction – Correct. A type 1 reaction, also known as a reversal reaction, is a type IV hypersensitivity reaction that can be seen in individuals with BT leprosy.^2^ This reaction may be due to the stronger cellular immune response found in BT leprosy cases. Symptoms of a type 1 reaction include erythema and edema in existing lesions, as well as neuritis.^2^ Notably, tuberculoid leprosy is not at risk for type 1 reactions.
B. A type 2 reversal reaction – Incorrect. A type 2 reaction, also known as erythema nodosum leprosum, can occur in patients with lepromatous (multibacillary) leprosy. This reaction presents with fever, painful nodules, and inflammation of various tissues.^2^
C. Lucio Phenomenon – Incorrect. Lucio phenomenon is a rare reaction that primarily occurs in untreated cases of LL and is characterized by severe necrotizing cutaneous lesions.^2^ This reaction is seen more commonly in specific regions of South and Central America.
D. Drug resistance – Incorrect. While drug-resistant strains of *Mycobacterium leprae* are increasing, patients with BT leprosy are more likely to develop a type 1 reaction. Furthermore, the use of multidrug therapy helps prevent the development of further drug resistance.^4^
E. None of the above – Incorrect. Although BT leprosy is considered the less severe form of leprosy and completely curable, complications can occur with proper treatment. Potential complications include immune-related responses and nerve damage.

## Conflicts of interest

None disclosed.
